# Development of KASP markers assisted with soybean drought tolerance in the germination stage based on GWAS

**DOI:** 10.3389/fpls.2024.1352379

**Published:** 2024-02-15

**Authors:** Qianru Jia, Miaomiao Zhou, Yawen Xiong, Junyan Wang, Donghe Xu, Hongmei Zhang, Xiaoqing Liu, Wei Zhang, Qiong Wang, Xin Sun, Huatao Chen

**Affiliations:** ^1^ Institute of Industrial Crops, Jiangsu Academy of Agricultural Sciences, Nanjing, China; ^2^ College of Life Science, Nanjing Agricultural University, Nanjing, China; ^3^ Japan International Research Center for Agricultural Sciences (JIRCAS), Tsukuba, Japan; ^4^ Zhongshan Biological Breeding Laboratory (ZSBBL), Nanjing, China

**Keywords:** soybean, drought tolerance, germination stage, GWAS, KASP

## Abstract

Soybean [*Glycine max*(L.)Merr.] is a leading oil-bearing crop and cultivated globally over a vast scale. The agricultural landscape in China faces a formidable challenge with drought significantly impacting soybean production. In this study, we treated a natural population of 264 Chinese soybean accessions using 15% PEG-6000 and used GR, GE, GI, RGR, RGE, RGI and ASFV as evaluation index. Using the ASFV, we screened 17 strong drought-tolerant soybean germplasm in the germination stage. Leveraging 2,597,425 high-density SNP markers, we conducted Genome-Wide Association Studies (GWAS) and identified 92 SNPs and 9 candidate genes significantly associated with drought tolerance. Furthermore, we developed two KASP markers for S14_5147797 and S18_53902767, which closely linked to drought tolerance. This research not only enriches the pool of soybean germplasm resources but also establishes a robust foundation for the molecular breeding of drought tolerance soybean varieties.

## Introduction

Soybean, which originated in China, has a cultivation history spanning over 3000 years (Sedivy et al., 2017; Li, 2021) and has evolved into a globally embraced crop due to its valuable composition. Soybean functions not only as a primary source of plant protein and oil for human consumption but also as fodder for animals (Zhao et al., 2021). Soybean also stands out for its unique contribution as a natural nitrogen fertilizer, thanks to its distinctive nodule structure (Sun et al., 2023).

Drought stress has always constrained the agriculture development and affects plant multiple physiological and biochemical indexes at all growth stages such as seed germination, seedling development, and flowering time (Zia et al., 2021; Poudel et al., 2023). Excessive drought disrupts photosynthesis, hampers metabolism, and ultimately jeopardizes crop survival (Ouyang et al., 2022). As global climate changes and human development, droughts will be more severe, frequent, and longer lasting in the future. Soybean is more sensitive to drought than other crops especially in germination stage (Zhao et al., 2022). Under dry or drought conditions, soybean yield may plummet by over 50%, highlighting the imperative to enhance soybean drought tolerance for food security (Arya et al., 2021; Cotrim et al., 2021). Given that seed germination marks the onset of plant growth and development (Waterworth et al., 2015), it is crucial to identify drought-tolerant genotypes during this stage for effective plant breeding.

There are numerous factors influence soybean seed germination, encompassing both external environmental conditions and internal seed factors. Among them, moisture is an important factor affecting germination. Exposure soybean to drought stress during germination stage can result in a 20% reduction in seedling numbers and a staggering 50% decrease in yield (Devi et al., 2014; Zhao et al., 2017). This poses a huge threat to agricultural production. Encouragingly, technological advancements now enable the screening of drought-tolerant varieties through methods such as molecular marker-assisted breeding and genetic modification.

With the rapid development of high throughput sequencing technology, genome-wide association studies (GWAS) have been widely used for plant genetic analysis. Notably, numerous single nucleotide polymorphisms (SNPs) and quantitative trait loci (QTLs) linked to drought tolerance in soybean have been identified through this approach (Hacisalihoglu et al., 2018). Recent investigations by Zhang et al. uncovered a significant drought-related locus on chromosome 16 (32,206,964 bp to 32,458,483 bp), and obtained a gene *Glyma.16G164400* (*GmPrx16*) associated with drought tolerance through haplotype analysis (Zhang et al., 2023). Saleem et al. conducted short- and long- duration drought experiments on a 359 soybean accessions at the seedling stage, and identifying 17 and 22 significant SNPs, respectively (Saleem et al., 2022). However, previous investigations into soybean drought tolerance primarily concentrated on seedling or mature stages, leaving uncertainty about whether the identified QTLs and SNPs exhibit similar adaptations during the germination stage. In contrast, Zhao et al. identified 26 SNPs on chromosomes 1, 4, 5, 6, 8, 9, 11, 15, and 20 associated with drought tolerance during the germination stage. Notably, this research utilized a multi-faceted evaluation approach, employing RGR (relative germination rate), RGE (relative germination energy), GDTI (germination drought tolerant index), GSI (germination stress index), and MFV (membership function value) as indicators (Zhao et al., 2022). However, due to differences of genetic background and environmental conditions, stable QTLs have not been detected under different environmental conditions, and valuable QTLs that could be further validated are also rare.

Building upon these advancements, our present study utilized Polyethylene glycol-6000 (PEG-6000) to modulate drought tolerance and determined the optimal treatment concentration through a concentration gradient. We assessed various germination traits, including germination rate (GR), germination energy (GE), germination index (GI), relative germination rate (RGR), relative germination energy (RGE), relative germination index (RGI), and average subordinative function value (ASFV) within a natural population of 264 Chinese soybean accessions during the germination stage. In addition, we conducted GWAS based on 2,597,425 high-density SNP markers, and identified SNPs significantly associated with drought tolerance. Subsequently, two KASP markers were successfully developed as a result of this comprehensive analysis. The overarching objective of this research is to screen and identify soybean germplasm resources that exhibit resilience to drought conditions.

## Materials and methods

### Plant materials and growth conditions

A natural population of 264 Chinese soybean accessions (with 212 improved varieties and 52 landraces) (Zhang et al., 2021) were used as the materials. All of the soybeans were planted in 2021 (E1) and 2022 (E2) in Nanjing, Jiangsu Province. Seeds in E1 and E2 were collected to conduct subsequent drought treatment experiments.

### Drought treatment and phenotypic determination

Dry soybean seeds with full grains, complete seed coat and uniform size were selected and disinfected with 2% NaClO2 for 15 min and then rinse three times with sterile water. Fifty of them put in a square with the size of 200 × 70× 70 mm. In each square, 30 g vermiculite is laid under the soybean seeds as a germination bed, and the surface of the seeds is covered with double layer filter paper to keep the surface of the seeds moist. A series of concentration gradients 0, 5%, 10%, 15%, 20% and 25% (w/v) of PEG-6000 (polyethylene glycol-6000) were used to simulate drought stress. After determining the optimal concentration, 60 mL of 15% (w/v) PEG-6000 was added to each soybean accession, and pure water treatment was used as the control. The temperature was set at 25 ± 1 °C, and the experiment was conducted under dark condition. Each processing is repeated three times. The germination was defined as when the length of the embryonic root extending beyond the hilum exceeding half of the longitudinal length of the seed,. The number of sprouts was counted daily. After the embryonic root breaks through the seed coat, 7 days is considered effective germination. GR, GE, GI, RGR, RGE and RGI were measured through the following formulas:


GR(%)=(Number of germinated seeds on the seventh day/total number of seeds)×100;

GE(%)=(Number of germinated seeds on the fourth day/total number of seeds)×100;

GI=Σ(Gt/Dt);

$$RGR=R_{D}/R_{C};$$

$$RGE=E_{D}/E_{C};$$

$$RGI=I_{D}/I_{C}.$$

In the above formulas, Gt is the number of germinated seeds per day, Dt is the number of germination days, D is the drought stress group, and C is the control group (Li et al., 2022).

Heritability (*h^2^*) calculation following the formula:


$$h^{2}=\sigma_{g}^{2}/\left(\sigma_{g}^{2}+\frac{\sigma_{ge}^{2}}{n}+\sigma^{2}/nr\right)$$

$\sigma_{g}^{2}$ (g=1,2,3… 264) is the genotype variance of the test material, $\sigma_{ge}^{2}$ (e=1,2) is the variance of the interaction between the genotype and the environment of the test material, *σ* ^2^ is the error variance, n is the number of environments, and r is the number of replicates (Knapp et al., 1985).

To calculate the ASFV, we used the following series of formulas calculations (Liu et al., 2005).

(1) Firstly, we calculated the drought tolerance coefficient (DTC) of each genotype;


$${\text{DTC}}_{ij}={\bar{y}}_{ij\left(treatment\right)}/{\bar{y}}_{ij\left(Control\right)}\times 100$$

${\bar{y}}_{ij\left(treatment\right)}$ and ${\bar{y}}_{ij\left(Control\right)}$ represent the j (j=1, 2, 3) trait average observed values of genotype i (i=1, 2, 3… 264) under drought and control treatment, respectively.

(2) We then standardized the DTC of each genotype using the subordinate function value (SFV);


$$F_{ij}=\left(DTC_{ij}-\min\left(DTCij\right)\right)/\left(\max\left(DTC_{ij}\right)-\min\left(DTC_{ij}\right)\right)$$

min(*DTC_ij_*) and max(*DTC_ij_*)represent the j (j=1,2,3) trait minimum and maximum DTC of genotype i (i=1,2,3… 264), respectively;

(3) Lastly, we calculated the average drought resistance coefficient of three drought- related traits, denoted as the ASFV.


$$A(F_{ij})=\frac{1}{3}\sum_{j=1}^{3}F_{ij}$$

Each genotype was assigned to a specific category corresponding to its level of drought tolerance, with the principle that a higher ASFV indicates a stronger drought tolerance of the genotype.

### GWAS

The SNP markers used for whole GWAS in soybean natural populations were derived from pre laboratory resequencing work, resulting in a high-density physical map encompassing a total of 2,597,425 SNPs (Zhang et al., 2021). GWAS was performed using the GAPIT package based on R software (Wang and Zhang, 2021). To mitigate the risk of false-positive associations, a mixed linear model (MLM) was implemented for GWAS (Wang et al., 2010). The significance threshold for identifying association sites was set at -Log_10_ (P) ≥ 5.0. Any SNP surpassing this threshold was deemed a significant association site.

### KASP

Genotyping was carried out using three sets of primers (F1, F2, and R) specifically designed for the KASP markers of S14_5147797 and S18_53902767 (**Supplementary Table S3**). These primers were designed using the Primer-Blast tool available on the NCBI website (https://www.ncbi.nlm.nih.gov/tools/primer-blast/index.cgi? LINK_LOC=BlastHome). Genomic DNA were extracted using the method of 2×CTAB (Mavrodiev et al., 2021). The PCR was amplified using the KASP V4.0 2×Mastermix (LGC, England), and the amplified procedure was conducted following the reagent instruction using a Quantitative Real-Time PCR System (ABI7500).

### Quantification and statistical analysis

P value, F value and degree of freedom were calculated by the IBM SPSS Statistics 25 software (IBM, Armonk, NY, USA). Means were compared using a one-way analysis of variance (ANOVA). The heritability was calculated using the lme4 package in R (http://www.Rproject.org/). A frequency distribution was created using Microsoft Excel 2021. To ensure statistical significance, analyses were performed in sufficiently large samples in all experiments.

## Results

### Screening of optimal concentration of PEG-6000

Six soybean germplasm (NPS36, NPS137, NPS140, NPS196, NPS213, NPS233) were selected randomly from the 264 Chinese soybean accessions. Germplasms underwent individual treatment with varying concentrations of PEG-6000, namely 0%, 5%, 10%, 15%, 20%, and 25% (w/v) PEG-6000 respectively. Six days later, the GR, GE and GI were assessed to identify the optimal concentration of PEG-6000. The results showed a consistent decline in GR, GE and GI with increasing concentrations of PEG-6000 for each soybean germplasms (**Supplementary Figures S1A, B**). Under 5% and 10% PEG-6000 treatment, GR, GE and GI exhibited no significant difference from the control (0%). However, under 20% and 25% PEG-6000 treatment, GR, GE and GI of some germplasms went to zero (**Supplementary Figures S1A–C**), rendering these concentrations unsuitable for further research. Only at the 15% PEG-6000 treatment did all GR, GE, and GI values exhibited a statistically significant difference compared to the control. Consequently, 15% PEG-6000 was identified as the optimal concentration for subsequent experiments.

### Descriptive statistics on the drought-tolerance traits of the 264 soybean accessions

This study investigated the drought-tolerance traits of soybean harvested in 2021 (E1) and 2022 (E2) during the germination stage. We treated the 264 soybean accessions with 0% (control group) and 15% PEG-6000 (drought treatment) and measured the GR, GE, and GI in two environments (**Table 1**). GRs of the 264 soybean accessions under drought treatment in E1 and E2 were 50.13% and 51.10%, while the controls were 88.55% and 83.90%, respectively. The coefficient of variations (CVs) of the GR under drought treatment were 49.20% and 41.69% while the controls were 11.56% and 13.49%, respectively. The GEs under treatment in E1 and E2 were 25.60% and 19.98%, while in the control conditions, they were 81.31% and 71.28%. The CVs of GE under drought treatment were 78.88% and 76.71%, while in the controls they were 19.26% and 21.44% in E1 and E2, respectively; the GIs under treatment were 9.87% and 6.33%, while the controls they were 2.82% and 2.51%, respectively. In E1 and E2, the CVs of GI after treatment were 61.18% and 54.27%, while the controls were 29.04% and 16.10%, respectively (**Table 1**). The three basic indicators of the control group were significantly greater than that under drought treatment, indicating that after 15% PEG-6000 drought treatment, the GR of soybean germplasms was greatly inhibited, leading to a decrease in GR, GE, and GI. At the same time, the CV of all three traits under drought treatment was significantly greater than that of control, indicating that GI, GR, and GE responses to drought vary significantly.

**Table 1:** Descriptive statistics of three germination-related traits of 264 accessions treated with 0% and 15% PEG-6000.

Trait	Treat		Max	Min	Range	Mean	SD	CV (%)
GR	E1	C	100.00.	41.67	41.67~100.00	88.55	10.24	11.56
		D	96.67	0.00	0.00~96.67	50.13	24.67	49.20
	E2	C	100.00	45.00	45.00~100.00	83.90	11.32	13.49
		D	90.00	0.00	0.00~90.00	51.10	21.30	41.69
GE	E1	C	100.00	33.33	33.33~100.00	81.31	15.66	19.26
		D	90.00	0.00	0.00~90.00	25.60	20.19	78.88
	E2	C	98.33	25.00	25.00~98.33	71.28	15.28	21.44
		D	75.00	0.00	0.00~75.00	19.98	15.33	76.71
GI	E1	C	3.861	17.111	3.86~17.11	9.87	2.87	29.04
		D	7.72	0.00	0.00~7.72	2.82	1.72	61.18
	E2	C	8.194	3.611	3.91~8.19	6.33	1.02	16 .10
		D	6.69	0.00	0.00~6.69	2.51	1.36	54.27

Moreover, a descriptive statistical analysis was conducted on RGR, RGE, RGI, and ASFV of 264 accessions. The average values of RGR, RGE, RGI, and ASFV in E1 and were 0.56, 0.30, 0.28, 0.40, 0.61, 0.28, 0.39 and 0.38, respectively (**Table 2**). The CVs of RGR were 47.00% and 42.11%, respectively. The CVs of RGR and RGI in E1 and E2 were 74.90%, 72.39%, 56.18% and 51.48%, respectively. The CVs of ASFV were 52.86% and 49.25%, surpassing the 50% threshold in both cases. These indicate that the population used in this study exhibits rich variation under drought conditions. Furthermore, the statistical analysis revealed heritability values of RGR, RGE, RGI, and ASFV were 49.82%, 44.31%, 44.14%, and 49.11%, respectively (**Table 2**). To a certain extent, it is beneficial for the screening of drought resistant soybean germplasm in germination stage.

**Table 2:** Descriptive statistics of germination-related traits of 264 accessions under drought stress.

Trait	Environment	Max	Min	Mean		SD	CV (%)	Skewness	Kurtosis	H^2^ (%)
RGR	E1	1.04	0.00	0.56	0.59	0.26	47.00	-0.231	-0.846	49.82
	E2	1.47	0.00	0.61		0.26	42.11	-0.026	-0.033	
RGE	E1	1.00	0.00	0.30	0.29	0.23	74.90	0.723	-0.146	44.31
	E2	0.88	0.00	0.28		0.20	72.39	0.684	-0.297	
RGI	E1	0.78	0.00	0.28	0.34	0.16	56.18	0.533	-0.031	44.14
	E2	0.97	0.00	0.39		0.20	51.48	0.265	-0.576	
ASFV	E1	0.94	0.00	0.40	0.39	0.21	52.86	0.206	-0.719	49.11
	E2	0.91	0.14	0.38		0.19	49.25	0.244	-0.579	

RGR, relative germination rate; RGE, relative germination energy; RGI, relative germination index; ASFV, average subordinative function value; SD, standard deviation; CV, coefficient of variation; H^2^, heritability.

The variation amplitude of GR, GE, and GI of the 264 soybean accessions in E1 surpassed that of E2, with each trait exhibiting a higher CV in E1 compared to E2. Notably, GR, GE, and GI displayed significant differences between E1 and E2, indicating that the drought tolerance traits of soybean germination stage may be selected during the process of variety improvement. Among the three drought tolerance traits, the RGR, RGE and RGI variation range of E1 and E2 spanned from 0.00~1.04, 0.00~1.47, 0~1.00, 0.00~0.88, 0.00~0.78 and 0.00~0.97, respectively (**Table 2**). It is evident that the variation range of RGI is comparatively smaller than that of RGR and RGE in E1 and E2. These indicated that the drought-tolerant traits influenced by environment in the soybean germination stage. Additionally, there were notable variations among the 264 soybean accessions.

### Variance and correlation analysis of drought tolerance traits across the 264 soybean accessions

We conducted a variance analysis on RGR, RGE and RGI of 264 soybean accessions in E1 and E2 (**Table 3**). The results showed that there were significant differences among genotypes, drought treatments and different environments. There are significant differences in genotype and environmental interaction effects between RGR and RGI, indicating that each genotype is influenced by environmental factors while experiencing differences in drought stress, and changes with environmental changes.

**Table 3:** Variance analysis of germination-related traits of 264 accessions under drought stress.

Trait	Variation sourse	DF	SS	MS	F value	P value
RGR	G	249	64.122	0.258	4.835	<0.05
	Env	1	0.479	0.479	8.995	<0.05
	G×Env	178	24.354	0.137	2.569	0.003
RGE	G	249	47.342	0.190	3.345	<0.05
	Env	1	0.073	0.073	1.287	<0.05
	G×Env	178	22.467	0.126	2.221	0.257
RGI	G	249	28.771	0.116	22.234	<0.05
	Env	1	1.317	1.317	253.360	<0.05
	G×Env	178	4.428	0.032	6.201	<0.05

G, germplasm; Env, environment; DF, degree of freedom; SS, sum of square; MS, mean square.

**Figure 1:**
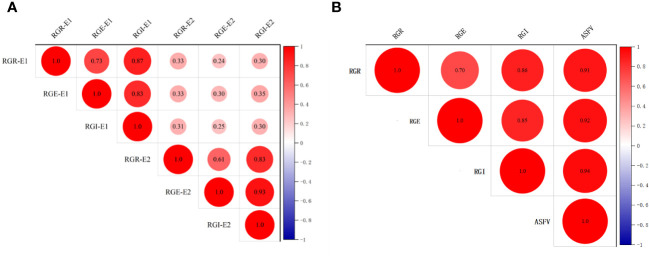
Correlation analysis of germination-related traits of 264 accessions. **(A)** Correlation analysis of RGR, RGE, RGI in E1 and E2. **(B)** Correlation analysis of RGR, RGE, RGI and ASFV.

To explore the correlation among RGR, RGE and RGI in E1 and E2, correlation analysis was conducted (**Figure 1**). The results showed that, the correlation of RGR, RGE and RGI of E1 and E2 is poor (**Figure 1A**). However, positive correlations were observed among RGR, RGE, RGI, and ASFV, with correlation coefficients of 0.70, 0.86 and 0.91, respectively (**Figure 1B**). In particular, RGE exhibited a significant positive correlation with RGI and ASFV, with correlation coefficients of 0.86 and 0.92, respectively. Additionally, a significant positive correlation between RGI and ASFV was identified, with a correlation coefficient of 0.94 (**Figure 1B**). These results illuminated the intrinsic connections among various drought-tolerant traits.

### Identification of drought tolerance grade and screening of drought-tolerance soybean germplasms

**Figure 2:**
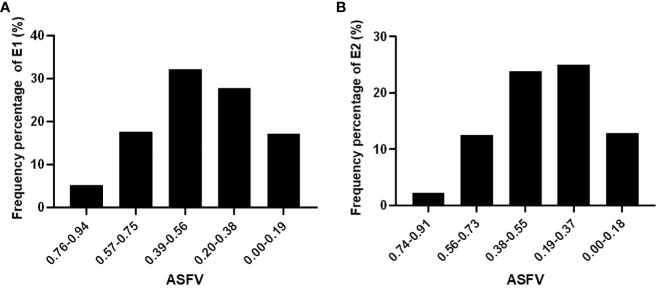
ASFV distribute frequency percentage in E1 **(A)** and E2 **(B)** environment.

This study standardized the RGR, RGE, and RGI of E1 and E2 using the subordinative function method. The ASFV of each genotype in the natural population was calculated, which were categorized into five groups to establish distinct drought resistance grading standards (**Figure 2**). These categories were graded as high drought tolerance (HDT), drought tolerance (DT), medium drought tolerance (MDT), drought sensitive (DS) and high drought sensitive (HDS), where higher ASFV values indicated stronger drought resistance within soybean germplasms.

In E1, the ASFV or the categories were defined as follows: HDT (above 0.75), DT (0.56-0.75), MDT (0.38-0.56), DS (0.19-0.38), and HDS (below 0.19) (**Figure 2A**). Notably, MDT soybean germplasms were the most abundant, constituting approximately 32% of the total, while DS germplasms ranked second, comprising 27%. Drought-resistant and drought-sensitive soybean germplasms were relatively balanced. (**Figure 2A**). HDT germplasm accounts for 5.29% of the total natural population of soybeans.

In E2, the ASFV for the defined categories were as follows: HDT (above 0.73), DT (0.5-0.73), MDT (0.37-0.55), DS (0.18-0.37), and HDS (below 0.18) (**Figure 2B**). Among them, DS soybean germplasms were the most prevalent, constituting approximately 25%, and the MDT germplasms ranked second, representing 23%. The number of drought-resistant soybean germplasms was comparable to that of DS germplasms. HDS germplasm accounted for 2.27% of the total soybean accessions.

This study successfully identified a total of 17 drought-tolerant soybean germplasms (**Table 4**), characterized by high GR, GE and GI. In E1, the HDT soybean germplasms included NX-F7-13, NX-F4-4, NX-F4-3, Guichundou 113, Zhonghuang 306, Guichundou 111, Gui26BC2-7, Huaidou 5, Huaidou 1, Doujiao 73, Xu 0701, and Jining 98-11497 (**Table 4**). In E2, the HDT soybean germplasms comprised Zhou 11019-2-1, Guichun 16, Gui0508-3, Huaidou 1, Nannong15-3, and Qihuang 35 (**Table 4**). Notably, Huaidou 1 demonstrated high drought tolerance in both environments. These identified HDT materials within soybean accessions serve as a valuable research foundation for further exploration into the genetic mechanisms of soybean drought resistance and as experimental resources for future soybean drought-resistant breeding.

**Table 4:** Drought-tolerant soybean germplasm screened in two environments.

Environment	High drought tolerance	ASFV
E1	NX-F7-13	0.79
	NX-F4-4	0.83
	NX-F4-3	0.79
	Guichundou 113	0.91
	Huaidou 5	0.82
	Huaidou 1	0.76
	Doujiao 73	0.80
	Xu 0701	0.80
	Jining 98-11497	0.94
	Zhonghuan 306	0.79
	Guichundou 111	0.79
	Gui 26BC2-7	0.86
E2	Huaidou 1	0.73
	Zhou 11019-2-1	0.74
	Guichun 16	0.78
	Gui 0508-3	0.78
	Nannong 15-3	0.75
	Qihuang 35	0.74

### GWAS signals related to six germination traits

We calculated the frequency of RGR, RGE, and RGI of 264 soybean accessions in E1 and E2 using Microsoft Excel 2021, and drew frequency distribution maps and density curves (**Supplementary Figure S2**). The absolute values of RGR, RGE, and RGI kurtosis and skewness are less than 1 (**Table 2**) and the histograms of phenotypic data exhibit an approximate normal distribution (**Supplementaryl Figure S2**), which indicated that the natural soybean population in this study has rich genetic variation and is suitable for subsequent genome-wide association analysis.

**Figure 3:**
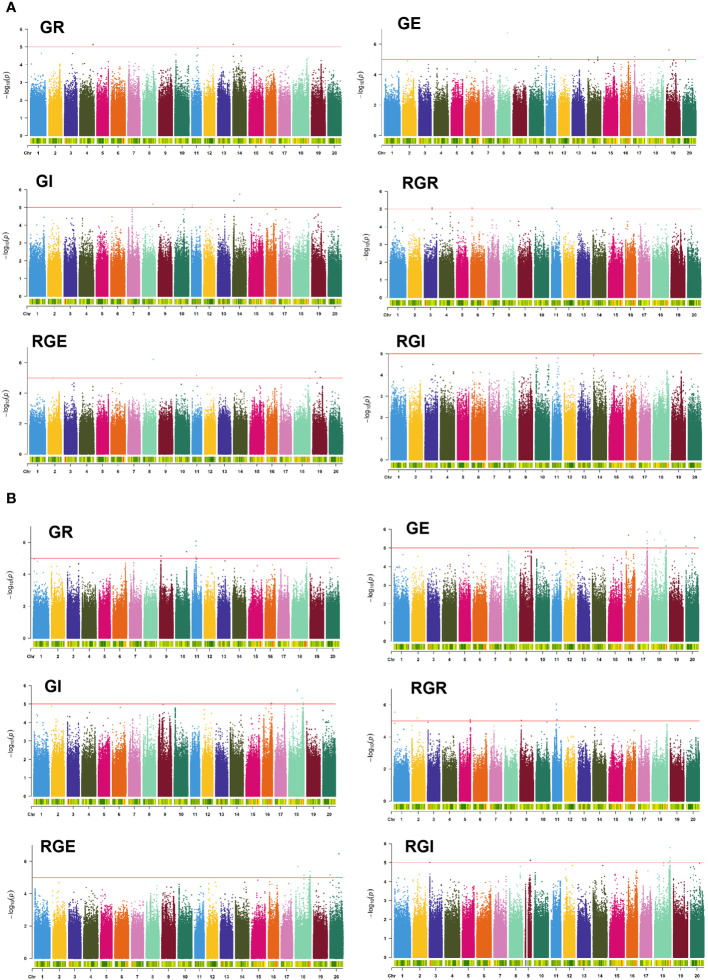
Manhattan plots for the GWAS for GR, GE, GI, RGR, RGE, and RGI in E1 **(A)** and E2 **(B)**. The red line indicates the significance threshold (−log_10_(*P*)=5.0).

To illuminate the genetic mechanism of six drought-tolerant germination traits, a GWAS with high-density SNPs was conducted to identify SNPs associated with six drought-tolerant germination traits. At a significance threshold of -Log_10_(*P*) ≥ 5.0, a total of 92 SNPs significantly associated with drought tolerance in germination stages were detected in this study, distributed across chromosomes 1, 2, 3, 4, 5, 6, 8, 9, 10, 11, 14, 16, 17, 18, 19 and 20 of the soybean genome (**Figure 3**, **Supplementary Tables S1**, **S2**).

Noteworthy SNPs included S08_37604504 locus on chromosome 8, significantly associated with GE, GI and RGE, explaining 10.82%, 10.08%, and 8.51% of the phenotypic variation, respectively (**Supplementary Tables S1**, **S2**). Additional SNPs like S18_32669137, S18_55108397 and S18_55108434 on chromosome 18 were significantly associated with the mentioned traits. S18_55108447 on chromosome 18 is significantly associated with GE, GI and RGE, explaining 10.2%, 10.07% and 10.2% of the phenotypic variation, respectively. On chromosome 9, S09_5096288 was significantly associated with GR and RGR, while S11_16409860 located on chromosome 11 was significantly associated with GE and GI. In addition, S11_2747816 and S11_2747823 on chromosome 11 were physically close to each other and related to RGE and RGI. S20_32847223 and S20_794406 located on chromosome 20, were significantly associated with GE and RGE. The SNPs significantly associated within 5543129~38193841 bp on chromosome 14 are all associated with two traits. The SNPs significantly associated with 35922040~36291124 bp on chromosome 17 are all related to GE.

### Identification of candidate genes of soybean drought-tolerant traits

**Figure 4:**
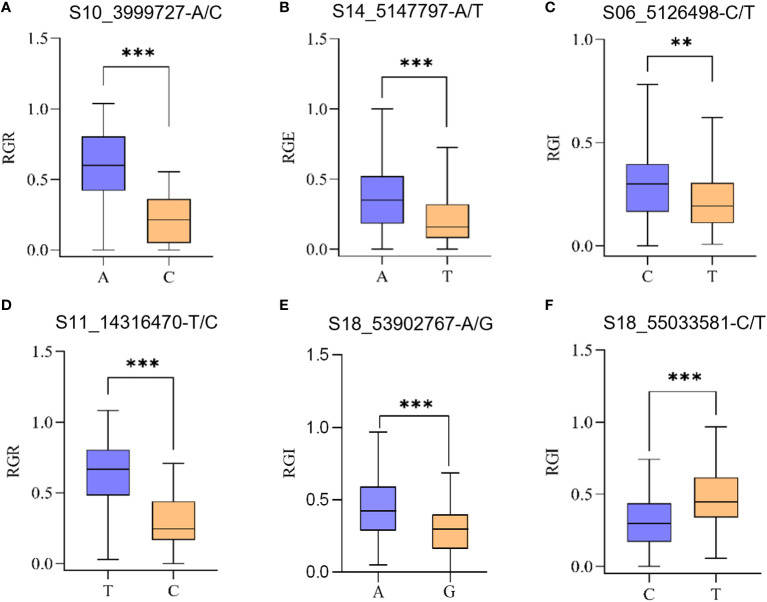
Significant SNP haplotype analysis of drought-related traits across 264 accessions. These SNPs were selected from the significant SNPs obtained from GWAS. **(A)** Average RGR of germplasms carrying S10_3999727-A/C; **(B)** Average RGE of germplasms carrying S14_5147797-A/T; **(C)** Average RGI of germplasms carrying S06_5126498-C/T; **(D)** Average RGR of germplasms carrying S11_14316470-T/C; **(E)** Average RGI of germplasms carrying S18_53902767-A/G; **(F)** Average RGI of germplasms carrying S18_55033581-C/T. The ** represents p<0.01 and *** represents p<0.001, one-way analysis of variance (ANOVA).

To investigate the phenotypic effects of allelic variations associated with significantly associated SNPs, haplotype analysis was conducted on SNPs surpassing the highest threshold. Notable findings include:

Allelic variation of S10_3999727 was A/C. Average RGR of germplasms carrying S10_3999727-A were higher compared to those carrying S10_3999727-C (**Figure 4A**).

Allelic variation of S14_5147797 was A/T, germplasms carrying S14_5147797-A exhibiting a significantly higher average RGE than those carrying S14_5147797-T (**Figure 4B**).

Allelic variation of S06_5126498 was C/T, germplasms carrying S06_5126498-C having a higher average RGI compared to those carrying S06_5126498-T (**Figure 4C**).

Allelic variation of S11_14316470 was T/C, germplasms carrying S11_14316470-C having lower RGR than those carrying S18_55033581-T (**Figure 4D**).

Allelic variation of S18_53902767 was A/G, average RGI of germplasms carrying S18_53902767-A were significantly lower than those carrying S18_53902767-T (**Figure 4E**).

Allelic variation of S18_55033581 was C/T, average RGI of germplasms carrying S18_55033581-C were significantly lower than that carrying S18_55033581-T (**Figure 4F**).

**Figure 5:**
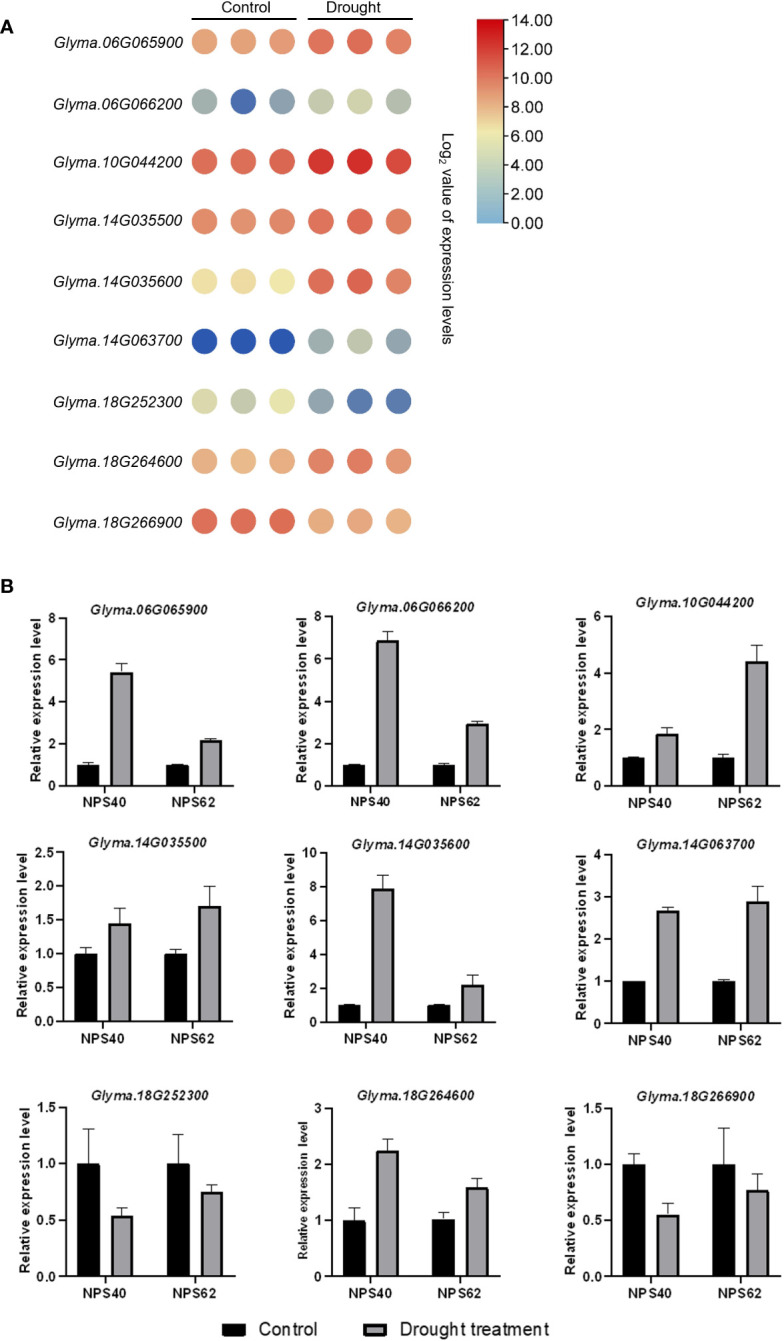
**(A)** Heat map of candidate genes relative expression levels under drought treatment. Each group has three replicates. One circle represents one value of the group. **(B)** Relative expression level of candidate genes of drought sensitive material NPS40 and drought tolerant material NPS 62.

In a previous study, we had conducted a linkage disequilibrium (LD) analysis on the soybean natural population in which the LD increased to 120 kb (Zhang et al., 2021). Referring to the gene functional annotation information of soybean, nine candidate genes significantly associated with drought tolerance during soybean germination stage were identified (**Table 5**). Including *Glyma.06G065900* (serine-rich protein-related protein), *Glyma.06G066200* (TOPLESS-related 1), *Glyma.10G044200* (Acyl-CoA N-acyltransferase with RING/FYVE/PHD-type zinc finger domain), *Glyma.14G035500* (vesicle-associated membrane protein 713), *Glyma.14G035600* (CRT (chloroquine-resistance transporter)-like transporter 3), *Glyma.14G063700* (HSP20-like chaperones superfamily protein), *Glyma.18G264600* (membrane-anchored ubiquitin-fold protein 2) and *Glyma.18G266900* (glycosyl hydrolase family 81 protein). Additionally, according to transcriptome data, all these genes exhibited altered expression levels under drought conditions, either induced or reduced (**Figure 5A**) (Wang et al., 2021b). To verify candidate genes expression level in drought resistant and drought sensitive material, we used the NPS 62 as drought resistant material and NPS40 as drought sensitive material. After treated with 15% PEG-6000 8 h, we sampled and conducted a qPCR. As is shown, some genes of NPS62 response to drought stress levels were higher than that of NPS40 (*Glyma.10G044200*, *Glyma.14G035500 and Glyma.18G264600*). But some genes of NPS62 response to drought stress levels were lower than that of NPS40 (*Glyma.06G065900*, *Glyma.06G066200* and *Glyma.14G035600*). Also, some of them between two materials didn’t show significant difference (*Glyma.14G063700, Glyma.18G252300 and Glyma.18G266900*) (**Figure 5B**).

**Table 5:** Candidate genes for drought-tolerant in the germination stage.

Gene ID	Homologs	Functional annotation
*Glyma.06G065900*	*AT5G11090*	serine-rich protein-related
*Glyma.06G066200*	*AT1G80490*	TOPLESS-related 1
*Glyma.10G044200*	*AT2G37520*	Acyl-CoA N-acyltransferase with RING/FYVE/PHD-type zinc finger domain
*Glyma.14G035500*	*AT5G11150*	vesicle-associated membrane protein 713
*Glyma.14G035600*	*AT5G53540*	CRT (chloroquine-resistance transporter)-like transporter 3
*Glyma.14G063700*	*AT1G53540*	HSP20-like chaperones superfamily protein
*Glyma.18G252300*	*AT2G44840*	Ethylene-responsive element binding factor 13
*Glyma.18G264600*	*AT5G15460*	membrane-anchored ubiquitin-fold protein 2
*Glyma.18G266900*	*AT5G15870*	glycosyl hydrolase family 81 protein

### Development of KASP markers of SNPs

**Figure 6:**
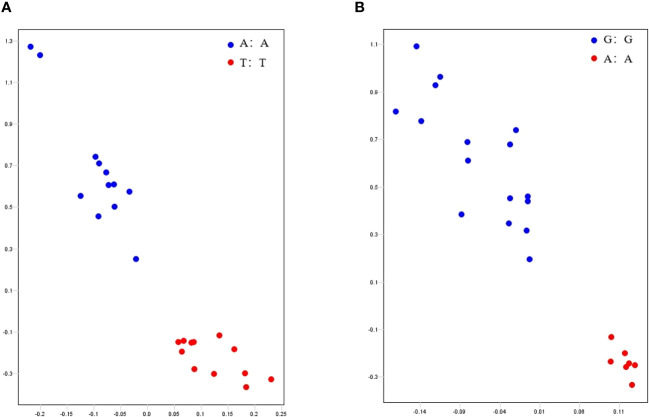
Genotyping of KASP markers. **(A, B)** were genotyping of S14_5147797 and S18_53902767, respectively. Blue and red dots in **(A)** represent the soybean germplasm carrying the A and T allele mutation sites, respectively. Blue and red dots in **(B)** represent the soybean germplasm carrying the G and A allele mutation sites, respectively.

Based on the GWAS results, we developed KASP markers for S14_5147797(A/T) and S18_53902767(A/G), which were significantly associated with RGE and RGI, respectively. Both of the genotypes can be clearly divided (**Figure 6**). In **Figure 6A**, blue dots represent soybean germplasm carrying the A allele mutation site, indicating higher RGE during the germination stage. Conversely, red dots represent soybean germplasm carrying the T allele mutation site, showing the opposite trend. In summary, soybean germplasm with the genotype AA generally exhibits a higher RGE than those with the genotype TT. In **Figure 6B**, blue dots represent soybean germplasms carrying the G allele mutation site, associated with a higher RGI during the germination stage. On the other hand, the red dot represents soybean germplasm carrying the A allele mutation site, correlated with a lower RGI. Overall, soybean germplasm with the genotype GG tends to have a higher RGI compared to those with the genotype AA. The distinct clustering of genotypes in these figures highlights the utility of the developed KASP markers for precise genotyping based on these significant SNPs, providing valuable information for further studies and breeding efforts targeting drought tolerance in soybeans during germination.

## Discussion

Drought tolerance remains a persistent challenge affecting crop growth, development, and yield. In the current study, we treated a natural soybean population of 264 accessions in the germination stage with15% PEG-6000 to modify the drought stress.

### Soybean germplasms resistance to drought tolerance in the germination stage

Numerous studies have addressed the identification of drought resistance traits during soybean germination, employing diverse soybean populations and proposing varying indicators, leading to nuanced definitions (Yang et al., 2013). The choice of drought resistance indicators significantly influences the authenticity and reliability of experimental outcomes. Zhang et al. (2005) advocated for a comprehensive approach, combining physiological, biochemical and morphological indicators to assess and select drought-resistant varieties. Wang et al. (2012) argued that soybean varieties exhibiting high GE also have high GR, with a strong correlation between their membership function values and the total membership function values. Consequently, in evaluating drought resistance during soybean germination, GR and GE can serve as valuable indicators. Commonly used indicators for drought resistance during the germination period encompass RGR, RGE, RGI, and germination drought tolerance index (GDTI), among others (Li et al., 2010). Recently, Zhao et al. used RGR, RGE, GDTI, GSI and membership function value (MFV) as the drought tolerance indicators, evaluating a natural soybean population of 410 accessions with 158,327 SNPs. In alignment with these considerations, our study adopts GR, GE, and GI as primary evaluation indicators for soybean drought resistance during germination. Recognizing the complex nature of soybean drought resistance, this research employs RGR, RGE, and RGI as additional indicators, integrating a membership function method for a comprehensive assessment of drought resistance within this population during germination. This approach facilitates a thorough comparison across different genotypes, employing equal interval divisions based on the ASFV range to establish standardized grading criteria for drought tolerance. By reducing subjective human grouping, this methodology enhances the demonstration of soybean responses to drought stress. Our analysis of phenotypic data for drought tolerance traits in 264 natural soybean accessions during germination reveals significant genotype variations post-drought stress treatment, indicating a degree of genetic stability. This affirms the suitability of this population for genetic screening of drought-resistant soybean germplasm.

This study categorized the natural soybean population into five distinct grades, ranging from HDT to HDS. A total of 17 soybean germplasms exhibiting HDT were selected (NX-F7-13, NX-F4-4, NX-F4-3, Guichundou 113, Zhonghuang 306, Guichundou 111, Gui26BC2-7, Huaidou 5, Huaidou 1, Doujiao 73, Xu 0701, Jining 98-11497, Zhou 11019-2-1, Guichun 16, Gui0508-3, Nannong 15-3 and Qihuang 35). Remarkably, Huaidou 1 demonstrated HDT in both environmental conditions (**Table 4**). The selected germplasms serve as valuable resources for future in-depth investigations into the molecular mechanisms of soybean drought resistance and genetic breeding endeavors. It is crucial to note that this experiment artificially simulated drought condition, the data and conclusions obtained may not entirely substitute conclusions drawn from field soil environments. Furthermore, the experiment specifically focuses on the germination stage, and the identified conclusions may not extend to other stages such as seedling and maturity.

### Candidate genes for resistance to drought tolerate in the germination stage

GWAS is widely used in the study of important agronomic traits. By effectively identifying SNP associated with the target trait, a total of 92 SNPs were associated with drought tolerance in the germination stage of this experiment (**Figure 3**, **Supplementary Tables S1**, **S2**). Unfortunately, the association SNPs identified in E1 and E2 did not co-locate with each other, perhaps indicating a lack of overlap between the two environments. This discrepancy may be attributed to variations in environmental conditions and other factors influencing the genetic associations with drought tolerance during germination.

Based on the GWAS results, nine candidate genes were identified: *Glyma.06G065900*, *Glyma.06G066200*, *Glyma.10G044200*, *Glyma.14G035500*, *Glyma.14G035600*, *Glyma.14G063700*, *Glyma.18G252300, Glyma.18G264600*, *Glyma.18G266900*. *Glyma.06G065900* is a serine-rich protein-related protein. Serine metabolism plays a crucial role in the plant’s response to various abiotic stresses (Kishor et al., 2020). When a plant is exposed to temperature, flooding, salt, drought or heat stress, serine accumulates (Stewart and Larher, 1980; Kaplan et al., 2004; Bocian et al., 2015; Hossain et al., 2017; Li et al., 2017). Transcriptome data also indicates an induction of *Glyma.06G065900* expression under drought stress (**Figure 5**). *Glyma.06G066200* codes a TOPLESS-related protein, TOPLESS (TPL) and TOPLESS-related protein (TPR) corepressors usually interact with transcription factors to regulate gene expression. TPRs have been known to influence hormonal signaling pathways, such as auxin, gibberellins, jasmonic acid and brassinosteroids, which are essential in plant stress responses (Saini and Nandi, 2022). Glyma.10G044200 is an Acyl-CoA N-acyltransferase with RING/FYVE/PHD-type zinc finger domain-containing protein. Acyl-CoA N-Acyltransferase involved in regulating plant meristem and architecture (Walla et al., 2020). Additionally, Acyl-CoA is the active metabolic intermediate in fatty acid synthesis and decomposition and potentially contributes to drought stress response through its involvement in lipid metabolism. *Glyma.14G035500* codes the protein vesicle-associated membrane protein 713. The exact role of this protein in drought response is not known, but vesicle-associated membrane proteins are generally involved in membrane trafficking, so it may be implicated in stress response pathways. From the web of TAIR (https://www.arabidopsis.org/index.jsp), we know that its homologous AtVAMP713 (AT1G53540) is involved in response to salt stress. A vesicle-associated membrane protein TaVAMP in wheat was identified as a drought-inducible protein, suggesting a potential role in abiotic stress tolerance (Singh et al., 2018). Glyma.14G035600 is a CRT-like transporter. In rice, OsCLT1 is a CRT-like transporter required for glutathione homeostasis and arsenic tolerance (Yang et al., 2016). The triple mutant *clt1 clt2 clt3* of Arabidopsis showed increased cadmium (Cd) sensitivity (Maughan et al., 2010). Further research is needed to determine whether it is involved in drought stress in soybean. Glyma.14G063700 and AT1G53540 (HSP17.6C) are homologous. It is involved in response to heat, hydrogen peroxide and salt stress (Yamaguchi et al., 2021; TAIR). *Glyma.18G252300* codes an ethylene-responsive transcription factor (ERF), which has been shown to be involved in responding to a wide range of abiotic stresses (Licausi et al., 2013; Xie et al., 2019). In wheat, TaERF87 and TaAKS1 synergistically regulate TaP5CS1/TaP5CR1-mediated proline biosynthesis to enhance drought tolerance (Du et al., 2023), while the transcriptome data reveals a reduction in the expression level of *Glyma.18G252300* in soybean under drought stress (**Figure 5**). Further experiments are required to elucidate the mechanisms underlying this observation. Glyma.18G264600 is a membrane-anchored ubiquitin-fold protein located in the plasma membrane. The plasma membrane is the primary site for sensing extracellular stimuli, and when cells are stimulated by abiotic stress, the cell membrane generates secondary signaling molecules such as reactive oxygen species (ROS) and phospholipids. Glyma.18G264600 may interact with second messengers to respond to drought stress. *Glyma.18G266900* is a glycosyl hydrolase family 81 protein, known to participate in responses to various stimuli such as bacterium, fungus, hormone-mediated signaling pathways, defense, osmotic stress, oxidative stress and water deprivation (TAIR). Glycosyl hydrolase family 1 (GH1) β-glucosidases in rice also responses to biotic and abiotic stress (Opassiri et al., 2006). When a plant is subjected to abiotic stress, the content of soluble sugars tends to increase. This elevation in soluble sugars has been associated with an enhancement in the plant’s stress resistance. In the case of soybeans under drought stress, the expression level of *Glyma.18G266900* was observed to decrease (**Figure 5**), This reduction in expression suggests a potential strategy employed by the plant to mitigate drought damage. It is conceivable that the plant decreases the expression of glycosyl hydrolase (*Glyma.18G266900*) to inhibit the hydrolysis of sugars, thus providing an additional protective mechanism against the adverse effects of drought stress.

In conclusion, the identified candidate genes are likely key contributors to soybean’s response to drought stress. Further comprehensive research is essential to unravel the specific functions of these genes in soybean drought tolerance, providing insights into their molecular mechanisms. This deeper understanding holds the potential to inform strategies for enhancing abiotic stress resistance in soybean varieties through targeted genetic improvements.

### Developing KASP markers

KASP (Kompetitive Allele-Specific PCR) marker technology holds significant importance and has wide applications in various fields, particularly in agriculture. Within agriculture, KASP markers are extensively used for multiple purposes, including germplasm resource identification, genetic relationship research, molecular marker-assisted breeding, genetic map construction and gene mapping. KASP has been applied to locate candidate genes for yield traits such as heading date, plant height, and thousand grain weight (Xiong et al., 2021; Xie et al., 2022); quality traits such as the color and shape of vegetables, fruits and other crops (Cheng et al., 2021; Shen et al., 2021); and genes of biotic and abiotic (Wang et al., 2018; Liu et al., 2020). In the current study, we developed two KASP markers S14_5147797 and S18_53902767 associated with drought tolerance (**Figure 6**). This achievement holds substantial application value for breeding drought-resistant soybean varieties. By using KASP markers, researchers and breeders can efficiently select and develop soybean varieties with improved drought tolerance, contributing to sustainable agriculture and food security.

The KASP markers developed in this study provide a valuable tool for soybean breeding programs aiming to enhance drought resistance.

## Data availability statement

The original contributions presented in the study are included in the article/**Supplementary Material**. Further inquiries can be directed to the corresponding authors.

## Author contributions

QJ: Data curation, Funding acquisition, Methodology, Writing – original draft, Writing – review & editing. MZ: Methodology, Software, Writing – review & editing. YX: Methodology, Software, Writing – review & editing. JW: Data curation, Methodology, Writing – review & editing. DX: Supervision, Writing – review & editing. HZ: Supervision, Writing – review & editing. XL: Supervision, Writing – review & editing. WZ: Methodology, Software, Supervision, Writing – review & editing. QW: Software, Supervision, Writing – review & editing. XS: Data curation, Investigation, Methodology, Supervision, Writing – review & editing. HC: Funding acquisition, Resources, Supervision, Visualization, Writing – review & editing.
