# Supplementary material for: Development of KASP markers assisted with soybean drought tolerance in the germination stage based on GWAS
Source: Front Plant Sci. 2024 Feb 15;15:1352379. doi: 10.3389/fpls.2024.1352379 (PMC10902137; doi:10.3389/fpls.2024.1352379)
Supplement: Supplementary file 1 [file DataSheet_1.pdf]

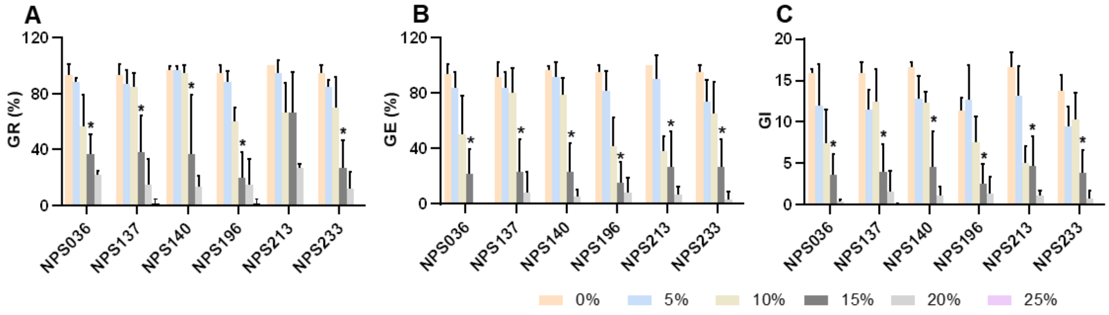

Supplemental Figure S1 Frequency distribution of six soybean germplasms GR (A), GE (B), GI (C). GR, germination rate; GE, germination energy; GI, germination index. NPS036, NPS137, NPS140, NPS196, NPS213, NPS233 were selected from the 264 accessions randomly. 0%, 5%, 10%, 15%, 20%, 25% (w/v) are the concentration of PEG-6000. Asterisks indicate significant differences from the control 0% PEG-6000 (***$P < 0.001$, **$P < 0.01$, and *$P < 0.05$). Three replicates and at least 100 seeds were counted for each treatment.

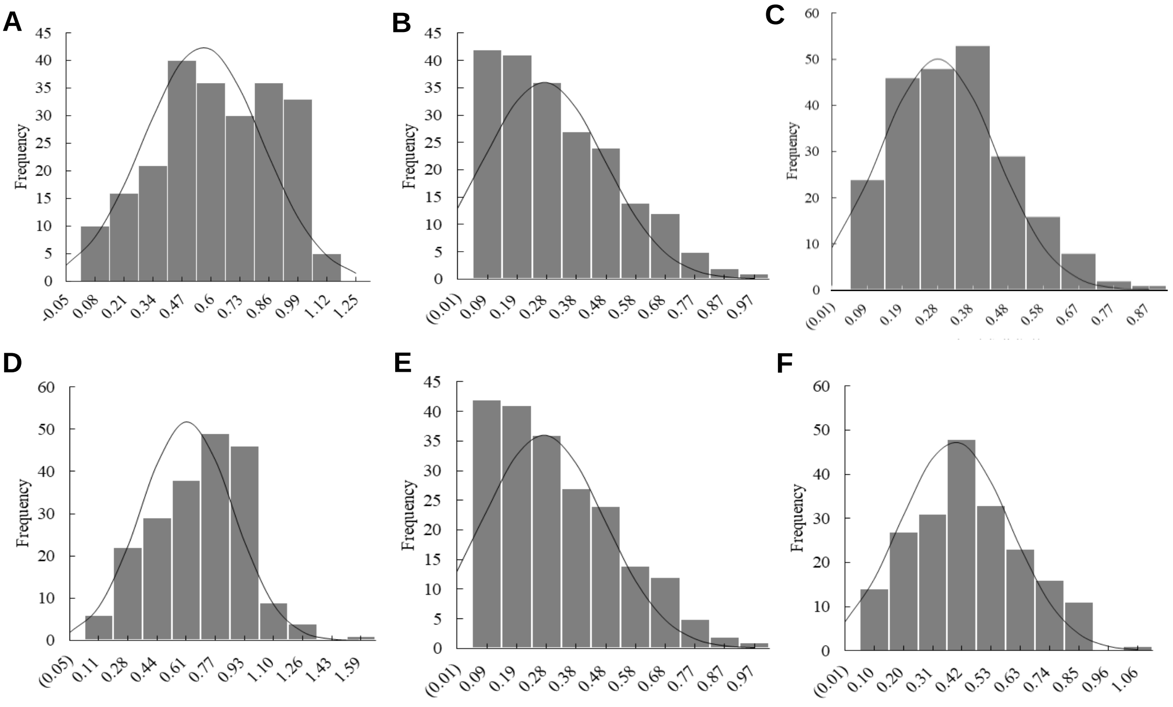

Supplemental Figure S2 The distribution of RGR, RGE, and RGI in the soybean natural population. (A-C) Frequency distributions of RGR, GRE and RGI in E1; (D-F) Frequency distributions of RGR, RGE, and RGI in E2
