# Supplementary material for: Development of KASP markers assisted with soybean drought tolerance in the germination stage based on GWAS
Source: Front Plant Sci. 2024 Feb 15;15:1352379. doi: 10.3389/fpls.2024.1352379 (PMC10902137; doi:10.3389/fpls.2024.1352379)
Supplement: Supplementary file 2 [file DataSheet_2.pdf]

Supplementary Table S1. Significant SNPs associated with GR, GE and GI.

Trait	Env.	Chr	SNP	Position	$-log_{10}(P)$	R^2（%）
GR	E1	4	S04_48057071	48057071	5.15	8.60
	E1	4	S04_48057074	48057074	5.13	8.56
	E2	9	S09_5096288	5096288	5.15	10.32
	E2	10	S10_40384427	40384427	5.42	10.96
	E2	11	S11_14316470	14316470	6.07	12.53
	E2	11	S11_14316501	14316501	5.79	11.86
	E2	11	S11_14308797	14308797	5.08	10.16
	E1	14	S14_2575905	2575905	5.15	8.61
GE	E1	8	S08_37604504	37604504	6.74	10.82
	E1	10	S10_34083831	34083831	5.17	7.93
	E1	11	S11_16409860	16409860	5.05	7.73
	E1	14	S14_38193836	38193836	5.15	7.90
	E1	14	S14_38193841	38193841	5.01	7.65
	E2	16	S16_14176559	14176559	5.69	11.59
	E1	17	S17_4798584	4798584	5.18	7.96
	E2	17	S17_36153962	36153962	5.86	12.00
	E2	17	S17_35922040	35922040	5.40	10.91
	E2	17	S17_36153961	36153961	5.22	10.48
	E2	17	S17_36291123	36291123	5.02	9.99
	E2	17	S17_36291124	36291124	5.02	9.99
	E2	18	S18_32669137	32669137	5.87	12.04
	E2	18	S18_32669176	32669176	5.77	11.79
	E2	18	S18_10688592	10688592	5.45	11.02
	E2	18	S18_51590433	51590433	5.42	10.95
	E2	18	S18_55108635	55108635	5.14	10.28
	E2	18	S18_55108434	55108434	5.10	10.20
	E2	18	S18_55108447	55108447	5.10	10.20
	E2	18	S18_55108397	55108397	5.10	10.20
	E2	18	S18_51596742	51596742	5.08	10.14
	E1	19	S19_10145745	10145745	5.61	8.74
	E2	20	S20_32847223	32847223	5.56	11.27
	E2	20	S20_794406	794406	5.10	10.19
GI	E1	8	S08_37604504	37604504	6.22	10.08
	E1	11	S11_16409860	16409860	5.16	8.10
	E2	16	S16_32444980	32444980	5.06	10.05
	E2	16	S16_34723248	34723248	5.03	9.98
	E2	18	S18_32669137	32669137	5.76	11.71
	E2	18	S18_32669176	32669176	5.68	11.53
	E2	18	S18_51590433	51590433	5.31	10.65
	E2	18	S18_55108397	55108397	5.07	10.07
	E2	18	S18_55108434	55108434	5.07	10.07
	E2	18	S18_55108447	55108447	5.07	10.07
	E1	19	S19_10145745	10145745	5.40	8.54
	E1	19	S19_27356988	27356988	5.02	7.85

Supplementary Table S2. Significant SNPs associated with RGR, RGE and RGI.

Trait	Env.	Chr	SNP	Position	$-log_{10}(P)$	R^2 (%)
RGR	E2	01	S01_3473137	3473137`	5.54	11.45
		02	S02_20306958	20306958	5.17	10.54
		05	S05_40686776	40686776	5.07	10.31
		09	S09_5096288	5096288	5.04	10.22
		11	S11_14316470	14316470	6.04	12.67
		11	S11_14316501	14316501	5.68	11.79
		11	S11_16832257	16832257	5.07	10.30
RGE	E1	8	S08_37604504	37604504	5.18	8.51
	E1	11	S11_2747816	2747816	5.15	8.45
	E1	11	S11_2747823	2747823	5.01	8.18
	E1	14	S14_25543129	25543129	5.75	9.62
	E1	14	S14_5147797	5147797	5.38	8.90
	E2	18	S18_10688592	10688592	5.67	11.72
	E2	18	S18_55033581	55033581	5.38	11.00
	E2	18	S18_32669137	32669137	5.14	10.43
	E2	18	S18_55036145	55036145	5.14	10.43
	E2	18	S18_55108635	55108635	5.13	10.41
	E2	18	S18_55033926	55033926	5.06	10.24
	E2	18	S18_55108434	55108434	5.04	10.20
	E2	18	S18_55108447	55108447	5.04	10.20
	E2	18	S18_55108397	55108397	5.04	10.20
	E2	18	S18_55036079	55036079	5.03	10.16
	E2	18	S18_55036161	55036161	5.01	10.12
	E2	20	S20_32847223	32847223	6.47	13.70
	E2	20	S20_794406	794406	5.16	10.49
RGI	E2	2	S02_142722	142722	5.70	11.76
	E2	3	S03_1857148	1857148	5.00	10.09
	E1	3	S03_28633053	28633053	5.07	9.07
	E1	3	S03_28633069	28633069	5.00	8.93
	E1	6	S06_5126498	5126498	5.06	9.05
	E2	9	S09_18126377	18126377	5.12	10.37
	E1	11	S11_2747816	2747816	5.02	8.96
	E1	11	S11_2747823	2747823	5.07	9.07
	E2	18	S18_53902767	53902767	5.75	10.05
	E2	18	S18_55033581	55033581	5.80	12.02
	E2	18	S18_55108550	55108550	5.27	10.73
	E2	18	S18_55108589	55108589	5.10	10.31
	E2	18	S18_55108625	55108625	5.27	10.73
	E2	18	S18_55108717	55108717	5.10	10.31
	E2	18	S18_55108734	55108734	5.10	10.31
	E2	18	S18_55108843	55108843	5.09	10.31
	E2	18	S18_55108860	55108860	5.10	10.31
	E2	18	S18_55108873	55108873	5.09	10.31
	E2	18	S18_55108874	55108874	5.10	10.31
	E2	18	S18_55108900	55108900	5.09	10.31
	E2	18	S18_55108901	55108901	5.09	10.31
	E2	18	S18_51540298	51540298	5.01	10.10
	E2	18	S18_55109348	55109348	5.08	10.27

Supplemental Table S3. Specific primers for KASP

		Sequence (5'-3')
S14_5147797	F1	GAAGGTGACCAAGTTCATGCTTTACTCATTACACCATGTC**A**
	F2	GAAGGTCGGAGTCAACGGATTACATTGCGAGGAGTGCTG**T**
	R	TGCAGTGATCTGATGGGAGA
S18_53902767	F1	GAAGGTGACCAAGTTCATGCTCTTATATGAATAAAATTTGA**A**
	F2	GAAGGTCGGAGTCAACGGATTCTTATATGAATAAAATTTGA**G**
	R	TGAAGGGAATAACAACTTTTAGCAA

Underlines in F1 indicate FAM fluorescent junction sequence and underlines in F2 indicated HEX fluorescent junction sequence.

Supplemental Table S4. Specific primers for qRT-PCR.

Genes		Sequence (5'-3')
Glyma.06g065900	F	GTATGTGCTCCCCTACGACG
	R	TTGATGCGAACTGGGACGAA
Glyma.06G066200	F	GGTTCACTGTTTGGGGTTGC
	R	AGCCAATGCAGTGTAGCCAT
Glyma.10G044200	F	AGACAACTGACGAATGGCGT
	R	TAAGGTTGACGCCTAGCAGC
Glyma.14G035500	F	CCGATGGACTCACCGTTCTC
	R	CGAGCTTGTTTCCTGAAGCG
Glyma.14G035600	F	GCTCAACTTGCCACTTTCGG
	R	CACCAGCTCCACTTGCTACA
Glyma.14G063700	F	TGTTCGGTGGTAGACGAAGC
	R	CTAATCTGCAGCACCCTCCC
Glyma.18G252300	F	GGTTTGGCTTGGCACGTATG
	R	ATCTGAACACGAGGACGACG
Glyma.18G264600	F	AGAGTGTTCTCGCTCAGTGG
	R	GACTCTGGCATTCCCCAACT
Glyma.18G266900	F	GCACAAACTTGGTGGCATGT
	R	CATCCCCTTCCACCCTTGAC
